# Fetal death due to upper airway compromise complicated by thyroid storm in a mother with uncontrolled Graves' disease: a case report

**DOI:** 10.1186/1752-1947-3-7297

**Published:** 2009-05-28

**Authors:** Recep Yildizhan, Mertihan Kurdoglu, Ertan Adali, Ali Kolusari

**Affiliations:** 1 Department of Obstetrics and Gynecology, Yuzuncu Yil University School of Medicine, Van, Turkey

## Abstract

**Introduction:**

We report an unusual case of upper airway compromise complicated by thyroid storm in a pregnant woman with Graves' disease, ending with the *in utero* death of the fetus. This complication might have developed due to upper airway edema as a result of poorly controlled hyperthyroidism.

**Case presentation:**

A 41-year-old Turkish woman at 27 weeks' gestation suffering from Graves' disease was referred to our emergency department with a diagnosis of respiratory arrest. She was unconscious and had been intubated. Her laboratory results were compatible with thyrotoxicosis. The patient had suffered from respiratory difficulty for a long time and had stopped using her antithyroid medications after the first trimester of pregnancy. One day before, she had visited an obstetrician because her respiratory distress had increased. At that time, her fetus was still alive. She was given oxygen therapy and then sent home. With a presumptive diagnosis of thyroid storm, she was admitted to the intensive care unit and treated with aggressive medical therapy. The baby was found to be no longer alive and was delivered vaginally after labor induction. The mother was discharged 10 days later with maintenance therapy.

**Conclusion:**

Hyperthyroidism during pregnancy warrants very close attention and should almost always be treated with appropriate antithyroid medications. Maternal respiratory distress in such patients can be an early sign of impending upper airway compromise and thyroid storm, which can endanger the mother and fetus unless prompt and aggressive therapy is initiated.

## Introduction

Thyrotoxicosis is a clinical syndrome caused by the circulation of excessive thyroid hormones and, if this is due to thyroid gland overactivity, it is called hyperthyroidism. Hyperthyroidism is one of the most common endocrine disorders in pregnancy (1 in 500 pregnancies), second only to diabetes [1]. The most common cause of thyrotoxicosis in women of childbearing age is Graves' disease (85% of all cases), which is an autoimmune condition mediated by stimulatory autoantibodies to the thyroid-stimulating hormone (TSH) receptor [2].

Findings associated with the normal hypermetabolic state of pregnancy can overlap with the signs and symptoms of thyroid disease. Most clinicians are aware of other signs and symptoms of hyperthyroidism that indicate thyroid disease and are not common in pregnancy, such as weight loss, hyperemesis, diarrhea, heart rate greater than 100/minute that does not decrease with the Valsalva maneuver, and/or lymphadenopathy [3]. However, upper airway edema is not traditionally considered a major risk to pregnant women with thyrotoxicosis and we are not aware that respiratory difficulty may be an early sign of this fatal complication.

We report an unusual case of upper airway compromise complicated by thyroid storm in a pregnant woman with undertreated Graves' disease, which resulted in respiratory arrest of the mother and death of the fetus.

## Case presentation

A 41-year-old Turkish woman pregnant for the fourth time with a past history of Graves' disease was referred to our emergency department with a diagnosis of respiratory arrest. She was unconscious and had been intubated. Her blood pressure and pulse rate were 160/90 mmHg and 120 beats/minute, respectively. Her body temperature was 36.5 °C. She also had a full goitrous thyroid gland with bilateral exophthalmos. From the history of the patient, it was learned that she had been diagnosed with Graves' disease one year before after consulting a general surgeon for respiratory difficulty and swelling of the neck. She was also positive for Pemberton's sign, which is the presence of facial plethora with both arms raised [4]. Her difficulty in breathing was thought to be due to her large goiter and a total thyroidectomy was planned for surgical treatment. She had started to use antithyroid drugs to become euthyroid before surgery. In addition, she was oligomenorrheic and did not know that she had conceived. She continued to use propylthiouracil 50 mg every six hours together with propranolol HCl 40 mg/day throughout the first four months of her pregnancy. Her respiratory difficulty resolved partially during that time. After she found out that she was definitely pregnant, she suddenly stopped taking her medications without consulting a physician and did not take them thereafter.

She was not followed regularly by an obstetrician during her pregnancy and was fine in the second trimester despite some mild respiratory problems. However, at the beginning of the third trimester, her respiratory difficulty worsened and one day before the respiratory arrest, she visited an obstetrician for respiratory distress. At that time, her fetus was still alive and found to be at the 27th week of gestation on sonography. She was given oxygen therapy and sent home. The next day, she was readmitted with severe respiratory distress together with stridor and she suffered respiratory arrest in the hospital. Using direct laryngoscopy, she was intubated with difficulty because of upper airway edema. After resuscitation, she was referred to us and her baby was found to be no longer alive. The patient was admitted to the intensive care unit for further evaluation and management. Initial maternal free triiodothyronine (T3), free thyroxin (T4), and thyroid-stimulating hormone (TSH) values were 17.6 pg/mL (1.80-4.71), 3.79 ng/dL (0.80-1.90), and 0.07µ IU/mL (0.400-4.0), respectively. Thyroglobulin was 184 ng/mL (0.73-84) while antithyroid peroxidase (TPO) antibody was 420 IU/mL (10-40) and antithyroglobulin antibody was 60 IU/mL (20-35). The patient was diagnosed with thyroid storm and treatment with propylthiouracil 150 mg every eight hours, propranolol HCl 40 mg/day, dexamethasone 0.5 mg/day, saturated solution of potassium iodide four drops every eight hours was started. An 1100 g female ex fetus was delivered vaginally after labor induction. After 48 hours in the intensive care unit, thyroid hormone levels started to decrease and she was extubated and transferred to our ward for further monitoring. The patient was discharged 10 days later with maintenance doses of propylthiouracil 200 mg every 8 hours and propranolol HCl 80 mg/day.

## Discussion

The natural course of Graves' disease is altered in pregnancy. The usual pattern is of aggravation in the first trimester and in the postpartum period with amelioration of symptoms in the second half of pregnancy [5]. An exception to this pattern is a subset of patients who present with long-standing hyperthyroidism, large goiters, or significant exophthalmos and, if they remain untreated, they may worsen in the last trimester and toxemia, cardiac failure, and even 'thyroid storm' may develop [6,7]. According to one study, there is evidence that pregnancy in some way precipitated thyrotoxicosis. In our case, since the patient continued to use her antithyroid medications in the first trimester, her symptoms were not aggravated. In the second half of her pregnancy, although she discontinued her medications, due to the natural silent course of the disease, no complications developed. However, at the beginning of the third trimester, the discontinuation of treatment resulted in the development of an upper airway obstruction together with thyroid storm.

Thyroid storm is a major risk to pregnant women with thyrotoxicosis and it most often occurs in undertreated or undiagnosed patients with another precipitating factor [3]. As many as 20% to 30% of cases can end in maternal and fetal mortality [8]. Maternal mortality is usually due to cardiac arrest [3] and most of the fetal morbidity and mortality are associated with significant obstetric complications including miscarriage (26%), low birth weight, prematurity, pre-eclampsia, and possibly congenital malformations [7].

Although it may have also been related to the thyroid storm, we speculate that respiratory arrest in our patient was due to upper airway compromise secondary to edema. Recently, Li Pi *et al.* published the only report stating that uncontrolled hyperthyroid patients with large goiters secondary to Graves' disease may develop edema of the upper airway [9]. Since the larynx and vocal cords of the patient were found to be edematous during the difficult intubation process, this may be regarded as the cause of the respiratory arrest. The presence of Pemberton's sign in this patient is also convincing evidence in favor of obstruction of venous and lymphatic drainage as a more likely cause of this patient's upper airway edema before pregnancy.

To the best of our knowledge, airway edema has not previously been reported as a cause of respiratory embarrassment in hyperthyroid pregnant women ending in fetal death. The fetal demise in our patient may be thought to be due to the effects of maternal TSH receptor antibodies acting on the fetal thyroid to cause fetal thyrotoxicosis and goiter [10]. The determination of TSH receptor antibodies (TSHRAb) or thyroid stimulating immunoglobulins (TSI) is indicated in mothers in whom previous pregnancies have been complicated by fetal or neonatal hyperthyroidism, in mothers with active disease on antithyroid drug therapy, in mothers with thyroidectomy during pregnancy, in mothers with a previous history of ablation therapy for Graves' hyperthyroidism, and in the presence of fetal tachycardia and incidental fetal goiter on ultrasonography. It is proposed that when serum TSI levels are more than 500% above normal values, after 24 to 28 weeks' gestation, the risk of fetal or neonatal hyperthyroidism is significant [2]. Since our patient was not followed regularly by an obstetrician and an endocrinologist during her pregnancy, these tests had not been performed before and we were also unable to perform these tests in our laboratory due to technical problems at that time. We also do not have any idea about fetal heart rate tracing just before fetal death since the fetus had died before the mother arrived at our hospital. However, we are sure that fetal tachycardia and fetal goiter were not detected on the detailed obstetric sonography performed the day before the event.

It has also been reported that the risk of fetal thyrotoxicosis is about 1% of all pregnancies in women with Graves' disease, and, if untreated, fetal mortality may be as high as 24%. Since our patient underwent obstetric sonography one day before her arrest, confirming the welfare of the fetus, we think the fetal demise was due to the collapse of umbilical circulation secondary to the maternal morbidity. The family of the fetus did not allow us to perform a necropsy of the fetus due to their religious beliefs; however, there was no mass in the anterior aspect of the fetal neck suggesting a goiter.

In the light of a previous report by Li Pi et al. [9], we also think that the respiratory difficulty in our patient may have also been due to the primary infiltrative process similar to Graves' ophthalmopathy and dermopathy potentially causing upper airway edema. Additionally, retrosternal goiter compression of venous drainage may have also contributed to the edema formation.

## Conclusion

Hyperthyroidism during pregnancy warrants very close attention and almost always should be treated with appropriate antithyroid medications. Maternal respiratory distress in uncontrolled pregnant patients can be an early sign of impending upper airway compromise and/or thyroid storm, which can endanger the mother and fetus unless prompt and aggressive therapy is initiated.

## Consent

Written informed consent was obtained from the patient for publication of this case report. A copy of the written consent is available for review by the Editor-in-Chief of this journal.

## Competing interests

The authors declare that they have no competing interests.

## Authors' contributions

RY was the primary consultant physician and was responsible for the management of the case. MK interpreted the patient data regarding the endocrinological disease and gestation. He also had primary responsibility for writing the manuscript. EA and AK contributed to this patient's evaluation and treatment. All authors read and approved the final manuscript.
